# Variation in the initial assessment and investigation for ovarian cancer in symptomatic women: a systematic review of international guidelines

**DOI:** 10.1186/s12885-019-6211-2

**Published:** 2019-11-01

**Authors:** Garth Funston, Marije Van Melle, Marie-Louise Ladegaard Baun, Henry Jensen, Charles Helsper, Jon Emery, Emma J. Crosbie, Matthew Thompson, Willie Hamilton, Fiona M. Walter

**Affiliations:** 1 The Primary Care Unit, Department of Public Health and Primary Care, University of Cambridge, Cambridge, UK; 2 Research Centre for Cancer Diagnosis in Primary Care, Research Unit for General Practice, Aarhus University, Aarhus, Denmark; 3 Julius Centre for Health Sciences and Primary Care, Utrecht University, Utrecht, Netherlands; 4 Centre for Cancer Research and Department of General Practice, University of Melbourne, Melbourne, Australia; 5 Gynaecological Oncology Research Group, Division of Cancer Sciences, University of Manchester, Manchester, UK; 6 Department of Family Medicine, University of Washington, Seattle, USA; 7 University of Exeter Medical School, University of Exeter, Exeter, UK

**Keywords:** Ovarian cancer, Cancer detection, Ovarian cancer symptoms, Ovarian cancer signs, Ovarian cancer tests, Cancer biomarkers, Symptom-triggered testing, Primary care, Clinical guidelines, Cancer pathways

## Abstract

**Background:**

Women with ovarian cancer can present with a variety of symptoms and signs, and an increasing range of tests are available for their investigation. A number of international guidelines provide advice for the initial assessment of possible ovarian cancer in symptomatic women. We systematically identified and reviewed the consistency and quality of these documents.

**Methods:**

MEDLINE, Embase, guideline-specific databases and professional organisation websites were searched in March 2018 for relevant clinical guidelines, consensus statements and clinical pathways, produced by professional or governmental bodies. Two reviewers independently extracted data and appraised documents using the Appraisal for Guidelines and Research Evaluation 2 (AGREEII) tool.

**Results:**

Eighteen documents from 11 countries in six languages met selection criteria. Methodological quality varied with two guidance documents achieving an AGREEII score ≥ 50% in all six domains and 10 documents scoring ≥50% for “Rigour of development” (range: 7–96%). All guidance documents provided advice on possible symptoms of ovarian cancer, although the number of symptoms included in documents ranged from four to 14 with only one symptom (bloating/abdominal distension/increased abdominal size) appearing in all documents. Fourteen documents provided advice on physical examinations but varied in both the examinations they recommended and the physical signs they included. Fifteen documents provided recommendations on initial investigations. Transabdominal/transvaginal ultrasound and the serum biomarker CA125 were the most widely advocated initial tests. Five distinct testing strategies were identified based on the number of tests and the order of testing advocated: ‘single test’, ‘dual testing’, ‘sequential testing’, ‘multiple testing options’ and ‘no testing’.

**Conclusions:**

Recommendations on the initial assessment and investigation for ovarian cancer in symptomatic women vary considerably between international guidance documents. This variation could contribute to differences in the way symptomatic women are assessed and investigated between countries. Greater research is needed to evaluate the assessment and testing approaches advocated by different guidelines and their impact on ovarian cancer detection.

## Background

Worldwide, ovarian cancer is the seventh most common cancer in women, with over 200, 000 new cases each year [1]. While once considered a silent killer, it is now recognised that symptoms occur in all stages of disease, although studies differ in the symptoms they report and the positive predictive value (PPV) they attribute to each symptom [2–5]. Given the modest PPVs of individual symptoms, e.g. 0.3% for abdominal pain and 2.5% for abdominal distension, symptoms alone cannot be used to diagnose ovarian cancer, but are routinely used to guide further assessment, including physical examination and testing [4].

An increasing range of tests are used in the initial investigation of symptomatic women for ovarian cancer, including the serum protein biomarker CA125 and imaging modalities such as transabdominal and transvaginal ultrasound, Computed Tomography (CT) and Magnetic Resonance Imaging (MRI). Algorithms that combine test results with patient characteristics such as age or menopausal state e.g. the Risk of Malignancy Index (RMI) and the ADNEX model, have also been developed to help predict ovarian cancer risk in women presenting with a pelvic mass [6, 7]. However, debate exists regarding the most accurate testing strategy for ovarian cancer. There is very limited research evaluating tests for the initial investigation of symptoms within the primary care setting [8, 9], where most women with this condition first present [10].

Given the discrepancies in the research literature on symptoms and the variety of testing options available, guidance documents, such as clinical practice guidelines, consensus statements and clinical care pathways, have been produced to aid clinicians in making practical decisions regarding the management of women with possible ovarian cancer. As these documents have the potential to significantly affect the care and healthcare outcomes for large numbers of patients, they should be rigorously developed, grounded in the evidence, and make unambiguous recommendations [11, 12].

In this review, we set out to systematically identify and assess the quality of international guidance documents covering the initial assessment for ovarian cancer in symptomatic women. In addition, we aimed to assess the consistency of guidance documents in terms of the symptoms and signs they include and the physical examinations and tests they recommend, to gain an insight into international variation in clinical practice.

## Methods

### Study selection

We selected documents that provided guidance on the initial assessment of women presenting with symptoms that might represent ovarian cancer i.e. an assessment conducted at the point at which women present with symptoms and enter a given healthcare system. As such, guidance documents that solely provided advice on investigation or management of women after a pelvic mass had been identified, a specialist referral made or a diagnosis of ovarian cancer given, were excluded. As this review focussed on guidance for women presenting with symptoms, the most common mode of ovarian cancer presentation [10, 13], documents which solely provided advice on screening of asymptomatic women or on the investigation of incidental pelvic masses, were excluded. Documents where guidance was limited to sub-groups of patients, e.g. hereditary cancer syndromes, were also excluded. Only documents produced by professional or governmental bodies and published within the ten years before 13th March 2018 were included. There were no language restrictions.

### Search strategy

Searches were conducted in Embase and MEDLINE. The MEDLINE search strategy is presented in Additional file 1**:** Figure S1. Additional searches were performed in guideline specific databases, namely, the National Guideline Clearing House, the Turning Research Into Practice (TRIP) database, the Guidelines International Network, the Canadian Partnership Against Cancer guidelines database, the Canadian Medical Association Infobase and the National Institute of Health and Care Excellence (NICE) website. All searches were performed between 1st and 13th of March 2018. The websites of more than 20 relevant international governmental and professional bodies were hand searched to supplement the database searches.

### Guideline selection

Two reviewers independently assessed titles and abstracts. Where either reviewer felt that a document met selection criteria or that it was not possible to exclude on the basis of title and summary alone, the full text was obtained and reviewed against the criteria. Disagreements were resolved by consensus.

### Data extraction

Two reviewers, fluent in the language of guideline publication, independently extracted data using a specifically developed template. Discrepancies in extraction were resolved by consensus.

Information on document characteristics (e.g. development body, year of development) and the process of development was collected. We classified documents into one of four categories, which best described their intended purpose and the development process, namely: (1) full Clinical Practice Guidelines (recommendations on patient care, informed by a systematic review of the evidence and taking account of benefits, harms and alternatives) [11]; (2) Short Guides (focused summary recommendations for patient care, not necessarily based on a full systematic literature review); (3) Consensus Statements (clinically relevant advice based on the opinion of an expert panel) [14], and (4) Clinical Pathways (a structured multidisciplinary plan of patient care, not necessarily based on a full systematic literature review) [15].

The healthcare system for which a guideline is developed will influence the recommendations. We applied a simplified version of the classification system developed by Bohm et al*,* categorising healthcare systems into three groups: National Health Service, National/Social Health Insurance and Private Health System [16].

Data relating to three components of the initial patient assessment were extracted: symptoms, physical examinations/signs, and investigations. Documents were categorised into the following five groups, based on the number of tests and the order of testing advocated: ‘single test’ i.e. one test advocated; ‘dual testing’ i.e. performing two tests concurrently; ‘sequential testing’ i.e. performing a second type of investigation (second line) if the first type of investigation (first line) is abnormal; ‘multiple testing options’ i.e. where a range of investigation options were presented with no single investigation being advocated above another; and ‘no testing’ i.e. where no specific tests were recommended as part of the initial assessment.

### Quality assessment

The AGREEII instrument was used to assess the quality of guidance development and reporting of included guidance documents [12]. This validated tool consists of 23 items divided into six domains: ‘Scope and Purpose’, ‘Stakeholder Involvement’, ‘Rigour of Development’, ‘Clarity of Presentation’, ‘Applicability’ and ‘Editorial Independence’. Each item is rated on a scale from one (criteria not met) to seven (criteria fully met). While developed for clinical practice guidelines, it has been used to assess other types of guidance document [14]. Two reviewers independently assessed each guidance document using the AGREEII tool. Assessments were compared and differences of three or more points per item were discussed and resolved by consensus. Combined scores for each domain were obtained using the following equation: (Obtained score – minimum possible score)/(maximum possible score – minimal possible score) × 100 [12]. We took a score of ≥50% in a particular domain to indicate ‘satisfactory’ quality [17].

## Results

### Guideline selection

**Fig. 1:**
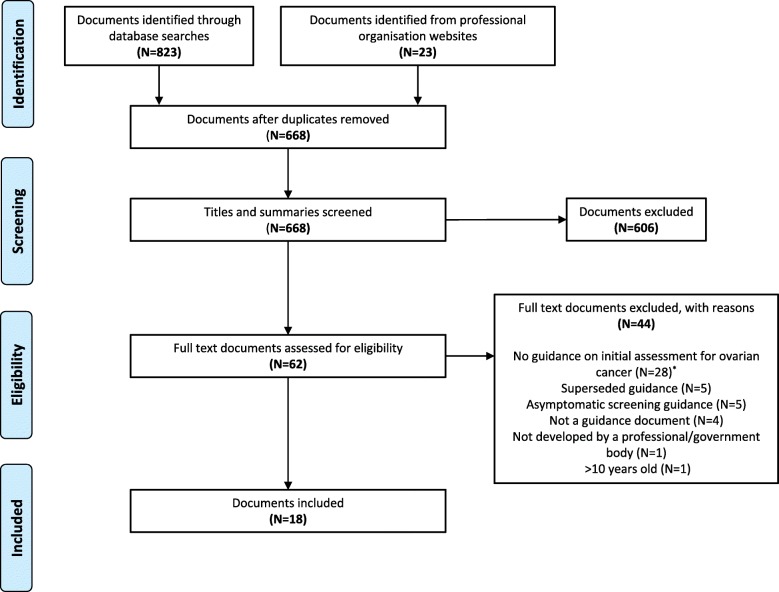
PRISMA flow diagram illustrating the document selection process. *Guidance covered the assessment/management of pre-identified pelvic masses (*N* = 11), other aspects of ovarian cancer e.g. treatment (*N* = 11) and cancers other than ovarian cancer (*N* = 6)

Our searches identified 846 documents, of which 178 were duplicates. The titles and summaries of 668 documents were screened, and 62 full text documents were obtained for further scrutiny. Eighteen documents met our selection criteria (Fig. 1).

### Guideline characteristics

Of the 18 documents that met the selection criteria, two were developed in continental Europe, five in the United Kingdom (UK) and Republic of Ireland, three in Scandinavia, four in North America and four in Australasia (Table 1) [18, 21–37]. Thirteen documents were published in English. Ten documents were categorised as full clinical practice guidelines, three as short guides, four as clinical pathways and one as a consensus statement. Documents varied in their intended audience and scope. Some dealt only with the initial assessment and referral of symptomatic patients and were aimed primarily at primary care practitioners [24, 26, 32–34]. Others also dealt with definitive diagnosis and treatment, often devoting more attention to this than initial assessment, and appeared to have a broader target audience including primary care practitioners and specialists [21, 22, 25, 29, 31, 35, 36]. Nine documents were developed for countries with National/Social Health Insurance Systems, seven for countries with National Health Services and two for a country with a Private Healthcare System.

**Table 1:** Characteristics of guidance documents presented by geographical area

Guidance document	Development body	Publication date of current version	Country and language if other than English	CPG	SG	CP	CS	Rigour of development (AGREEII) %	Healthcare system
Continental Europe									
Epithelial ovarian carcinoma	Dutch Society for Obstetrics and Gynaecology (NVOG)	2018	Netherlands (Dutch)	♦				66	National/Social Health Insurance
Guideline on diagnostics, therapy and follow-up of malignant ovarian tumours	The Association of Scientific Medical Societies in Germany (AWMF), led by German Society for Gynaecology and Obstetrics (DGGG)	2017	Germany (German)	♦				81	National/Social Health Insurance
United Kingdom and Republic of Ireland									
Epithelial ovarian / fallopian tube / primary peritoneal cancer guidelines: recommendations for practice	British Gynaecological Cancer Society	2017	UK	♦				48	National Health Service
Ovarian cancer GP referral for symptomatic women	National Cancer Control Programme	2016	Republic of Ireland		♦			7	National/Social Health Insurance
Suspected cancer: recognition and referral	National Institute for Health and Care Excellence (NICE)	2015	England, Wales, Northern Ireland	♦				96	National Health Service
Scottish referral guidelines for suspected cancer	Healthcare Improvement Scotland	2014	Scotland	♦				55	National Health Service
Management of epithelial ovarian cancer	Scottish Intercollegiate Guidelines Network (Part of Healthcare Improvement Scotland)	2013	Scotland	♦				76	National Health Service
Scandinavia									
Integrated ovarian cancer patient pathway	The Danish National Health Authority	2016	Denmark (Danish)			♦		29	National Health Service
Ovarian cancer patient pathway	The Norwegian Directorate of Health	2016	Norway (Norwegian)			♦		38	National Health Service
Standardised ovarian cancer care pathway ^a^	Regional Cancer Centre Co-operative Sweden	2015	Sweden (Swedish)	♦				55	National Health Service
Australasia									
Assessment of symptoms that may be ovarian cancer: a guide for general practitioners^b^	Cancer Australia	2015	Australia		♦			50	National/Social Health Insurance
Appropriate referral of women with suspected ovarian cancer^b^	Cancer Australia	2015	Australia		♦			50	National/Social Health Insurance
Optimal care pathway for women with ovarian cancer	Cancer Council Victoria	2015	Australia			♦		10	National/Social Health Insurance
Suspected cancer in primary care: Guidelines for investigation, referral and reducing ethnic disparity	New Zealand Guidelines Group	2009	New Zealand	♦				56	National/Social Health Insurance
North America									
Ovarian cancer: including fallopian tube cancer and primary peritoneal cancer	National Comprehensive Cancer Network	2018 (v2)	USA	♦				65	Private Health System
The role of the obstetrician-gynaecologist in the early detection of epithelial ovarian cancer in women at average risk	American College of Obstetrician Gynaecologists and the Society of Gynaecological Oncology	2017	USA				♦	11	Private Health System
Ovarian cancer diagnosis pathway map	Cancer Care Ontario	2016	Ontario, Canada			♦		19	National/Social Health Insurance
Genital tract cancers in females: ovarian, fallopian tube, and primary peritoneal cancers	Guidelines and Protocol Advisory Committee (Medical Services Commission)	2014	British Columbia, Canada	♦				16	National/Social Health Insurance

*CPG* Clinical Practice Guideline, *SG *Short Guideline, *CP *Clinical Pathway, *CS* Consensus Statement

^a^A full clinical practice guideline covering initial assessment, definitive diagnosis and treatment [18], and a short version focussing on initial assessment and investigation in primary care [19], are available. Guidance on initial assessment differed slightly between the two documents. The recommendations presented in this review were extracted from the short guide. AGREEII appraisal included an assessment of the full guideline evidence review

^b^Short guide, still active. Based on a now rescinded 2004 full clinical practice guideline entitled ‘Clinical practice guidelines for the management of women with ovarian cancer’ [20]. AGREEII appraisal included an assessment of the full guideline evidence review

### Quality assessment

Two guidance documents scored ≥50% in all six domains (Additional file 1: Table S1). Scores for the Rigour of Development domain (which appraises the process of evidence identification, synthesis, assessment and recommendation formulation) ranged from 7 to 96%, with 10 documents scoring ≥50% (Table 1).

### Symptoms

**Fig. 2:**
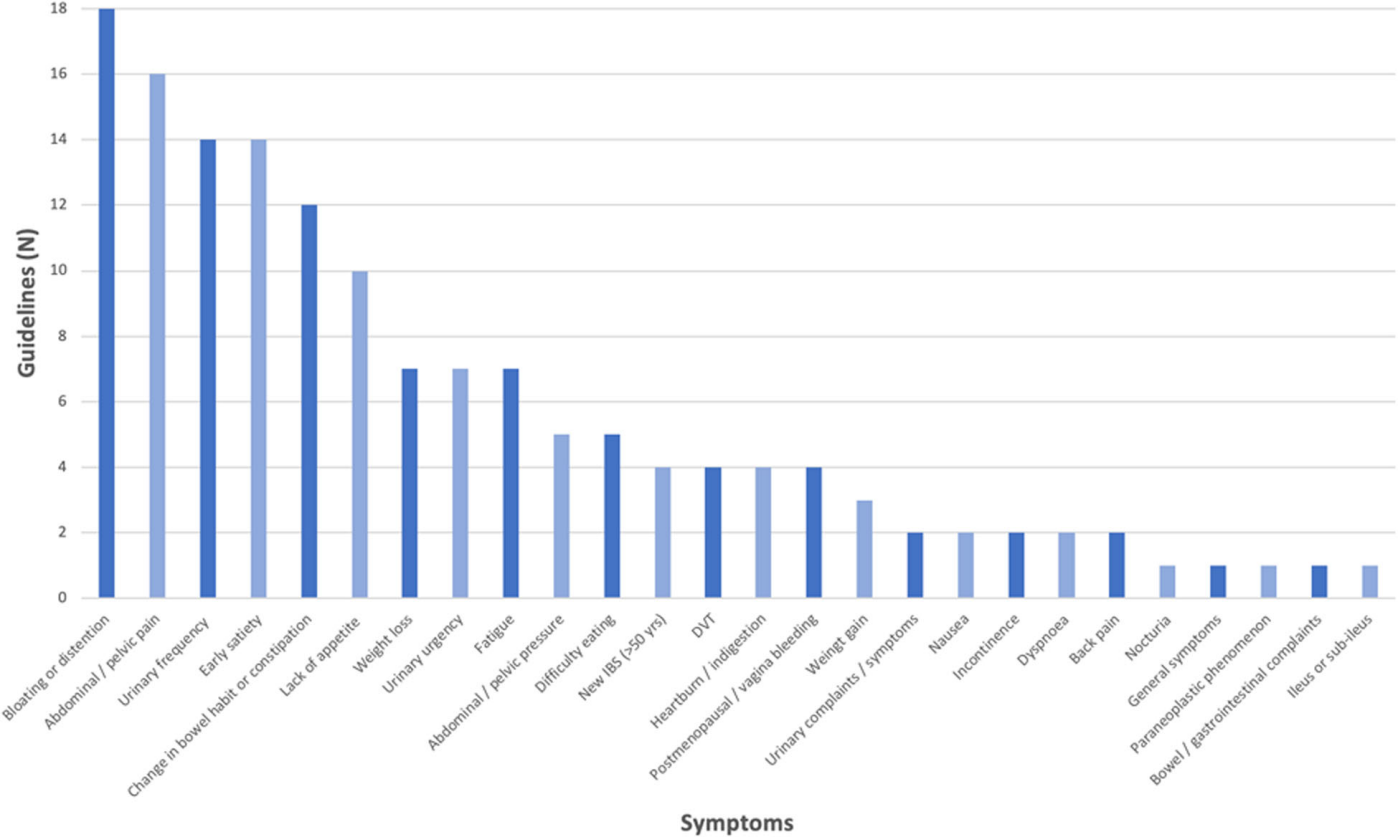
Symptoms included in guidelines

All guidance documents provided advice regarding presenting symptoms that should prompt a doctor to consider ovarian cancer. The numbers of guidelines in which each symptom was included is shown in Fig. 2. One or more of the related terms bloating, abdominal distention, increased abdominal size or girth, were listed as symptoms of ovarian cancer in all documents, abdominal or pelvic pain in 16 documents, urinary frequency in 14 documents and feeling full or early satiety in 14 documents. We identified 20 symptom terms that were included in under 50% of documents. The number of symptom terms included in the recommendations of documents ranged from four to 14 (Additional file 1: Table S2). Some documents simply listed symptoms doctors should be aware of in relation to ovarian cancer, while others provided further details on symptom frequency (e.g. > 12x/month), nature (e.g. persistent), duration (e.g. > 1 year) and age at presentation (e.g. > 50 years).

### Physical examinations and signs

Fourteen documents provided guidance on physical examination or the signs associated with ovarian cancer (Table 2). Thirteen of these documents specifically advocated abdominal examination or mentioned abdominal signs. Nine documents specifically advocated pelvic or gynaecological examination, three of which detailed that this should include a speculum examination, three a bimanual or digital examination and one a vaginal examination, while three documents recommended a rectal examination.

**Table 2:** Physical examinations recommended and ovarian cancer signs noted within guidance documents

Document	Type of examination specified	Signs
Continental Europe		
Epithelial ovarian carcinoma (Neth)	Not specified	- Pelvic mass / abdominal mass
		- Ascites
		- Pleural effusion
		- Increased uterine / vaginal prolapse
		- Enlarged supraclavicular lymph nodes
Guideline on diagnostics, therapy and follow-up of malignant ovarian tumours (Ger)	Abdominal and pelvic / gynaecological examination (including digital and speculum)	- Ovarian mass
United Kingdom and the Republic of Ireland		
Epithelial Ovarian / Fallopian Tube / Primary Peritoneal Cancer Guidelines: recommendations for practice (UK)	Examination	- Pelvic or abdominal mass
Suspected cancer: recognition and referral (Eng)	Physical examination	- Ascites
		- Pelvic / abdominal mass (not obviously uterine fibroids)
Ovarian cancer GP referral for symptomatic women (Ire)	Clinical examination (include a bimanual-pelvic examination)	- Unexplained ascites
		- Pelvic mass
		- Palpable ovaries in postmenopausal women
Scottish referral guidelines for suspected cancer (Scot)^a^	Abdominal palpation	- Ascites
		- Pelvic or abdominal mass (not obviously uterine fibroids, gastrointestinal or urological in origin)
Management of epithelial ovarian cancer (Scot)	Not specified	- Not specified
Scandinavia		
Integrated ovarian cancer patient pathway (Den)	Gynaecological examination (including palpation and speculum)	- Ascites
		- Pelvic mass
Ovarian cancer patient pathway (Nor)	Not specified	- Not specified
Standardised ovarian cancer care pathway (Swed)^b^	Palpation of superficial lymph nodes, abdominal palpation, rectal examination and auscultation of the heart and lungs	- Pleural effusion (unexplained)
		- Ascites
Australasia		
Assessment of symptoms that may be ovarian cancer: a guide for general practitioners (Aus)	Abdominal palpation, pelvic assessment, vaginal and rectal examination	- Firm resistance on abdominal palpation
		- Unexplained fullness
		-Fullness + shifting dullness on percussion
		- Hard irregular mass in the pouch of Douglass
		- Adnexal mass
Appropriate referral of women with suspected ovarian cancer (Aus)	Not specified	- Not specified
Optimal care pathway for women with ovarian cancer (Aus)	General and pelvic examination	- Not specified
Suspected cancer in primary care: guidelines for investigation, referral and reducing ethnic disparity (NZ)	Abdominal palpation and pelvic examination	- Not specified
North America		
Ovarian cancer: including fallopian tube cancer and primary peritoneal cancer (USA)	Abdominal and pelvic examination	- Suspicious palpable pelvic or abdominal mass
		- Ascites or abdominal distension
The role of the obstetrician-gynaecologist in the early detection of epithelial ovarian cancer in women at average risk (USA)	Not specified	- Not specified
Ovarian cancer diagnosis pathway map (Ont, Can)	Directed physical examination. Pelvic examination including speculum and bimanual examinations and examination of the external genitalia	- Suspicious palpable pelvic or abdominal mass
		- Ascites
Genital tract cancers in females: ovarian, fallopian tube, and primary peritoneal cancers (BC, Can)	A physical examination of the abdomen and pelvis including a pelvi-rectal examination	- Abdominal mass

^a^As recorded on associated Microsite and Short guidance document. The full guideline covers all gynaecological cancers with examinations and findings listed together. Microsite and Short guideline lists examinations and signs by cancer site

^b^Both a full clinical practice guideline covering initial assessment, definitive diagnosis and treatment, and a short version focusing on initial assessment and investigation in primary care, are available. Guidance on initial assessment differed slightly between the two documents. The presented data was extracted from the short guide

### Tests

Fifteen documents provided advice on the initial investigation of symptoms and were categorised based on the number and order of tests recommended (Table 3). One document advocated a single test strategy, four a duel testing strategy, four a sequential testing strategy, three gave multiple testing options, and three did not advocate testing prior to referral, although two of these did recommend that a CA125 sample be taken at the point of specialist referral so as to be available to the specialist. One document could not be categorised as it was unclear when and how tests should be used in the initial assessment for ovarian cancer [21]. The most commonly advocated tests for initial investigation were CA125 (11 documents) and ultrasound (12 documents). Several guidelines also recommended using additional cancer biomarkers such as CA19–9, CEA, AFP and HCG, routine blood tests including full blood count and renal function, imaging tests including CT and MRI, and the risk tools RMI and ADNEX.

**Table 3:** Summary of tests recommended for the assessment of symptoms and/or signs of ovarian cancer

Strategy	Guideline	When is testing advocated?	Initial tests
Single test	Guideline on diagnostics, therapy and follow-up of malignant ovarian tumours (Ger)	Signs or symptoms of ovarian cancer (OC)	Transvaginal US *Note:* *CT, MRI, PET CT may be used in specific cases*
Dual testing	Scottish referral guidelines for suspected cancer (Scot)	Symptoms of OC *Note:* *Ascites- refer urgently rather than test*	CA125 + pelvic US
	Management of epithelial ovarian cancer (Scot)	Symptoms of OC	CA125 + pelvic US
	Assessment of symptoms that may be ovarian cancer: a guide for general practitioners (Aus)	Mass identified clinically *Note:* *No mass identified clinically- refer appropriately*	CA125 + transvaginal US *Or* CA125 + Abdominal US *Or* CA125 + CT
	Appropriate referral of women with suspected ovarian cancer (Aus)	Suspicious findings on clinical examination	CA125 + transvaginal US +/− calculation of Risk of Malignancy Index (RMI)
Sequential testing	Suspected cancer: recognition and referral (Eng)	OC symptoms *Note:* *Ascites or suspicious mass- refer urgently rather than test*	First line: CA125 Second line: Abdominopelvic US (if CA125 is abnormal)
	Epithelial ovarian / fallopian tube / primary peritoneal cancer guidelines: recommendations for practice (UK)	OC symptoms *Note:* *Pelvic or abdominal mass- refer urgently rather than test*	First line: CA125 Second line: Abdominopelvic US (if CA125 is abnormal)
	Ovarian cancer GP referral for symptomatic women (Ire)	History suspicious of OC but examination normal *Note:* *Suspicious pelvis mass or ascites- refer urgently rather than test*	First line: CA125 Second line: US of pelvis (If CA125 35–200 u/ml) *Note:* If CA125 > 200 u/ml refer without US
	Ovarian cancer diagnosis pathway map (Ont, Can)	Suspicion of OC *Note:* *Tests may be performed prior to specialist referral but are not a requirement for referral. Can refer prior to testing*	First line: Transvaginal US and / or other imaging Second line: CA125, FBC, Renal Function + RMI *(*If indicated: *CEA, CA19–9, other tumour markers* e.g. *AFP, LDH, HCG)*
Multiple testing options	Optimal care pathway for women with ovarian cancer (Aus)	Symptoms of OC	Pelvic US + Routine blood tests + CA125 + Algorithms such as RMI, ADNEX +/− CT scan
	Genital tract cancers in females: ovarian, fallopian tube, and primary peritoneal cancers (BC, Can)	Suspicion of OC *Note:* *Imaging not essential for referral*	Transvaginal or abdominal US Blood tests: CA125***,*** CA19–9***,*** CA15–3, CEA *< 40 yrs old:* AFP, HCG, LDH
	Ovarian cancer Including fallopian tube cancer and primary peritoneal cancer (USA)	Suspicion of OC *Note:* *Provides some advice on when particular tests are indicated. Appears to include both initial and pre-surgical tests*	US *and/or* abdominal/pelvic CT/MRI (as indicated) Chest CT or chest x-ray (as indicated) Complete blood count, chemistry profile and LFT CA125 or other tumour markers (as indicated: inhibin, β-hCG, AFP, LDH, CEA, CA19–9) Nutritional status GI evaluation (as indicated)
No testing prior to referral	Integrated ovarian cancer patient pathway (Den)	At point of specialist referral	*Note* *CA125 requested in primary care at time of referral so as to be available to the specialist. Not acted upon in primary care*
	Ovarian cancer patient pathway (Nor)	Post specialist referral	Post referral
	Standardised ovarian cancer care pathway (Swed)	At point of specialist referral	*Note* *CA125 requested in primary care at time of referral so as to be available to the specialist. Not acted upon in primary care*
Unclear or no recommendations on testing given	Suspected cancer in primary care: guidelines for investigation, referral and reducing ethnic disparity (NZ)	No recommendations	No recommendations
	The role of the obstetrician-gynaecologist in the early detection of epithelial ovarian cancer in women at average risk (USA)	No recommendations	No recommendations
	Epithelial ovarian carcinoma (Netherlands)	Suspicion of OC. Not clear which tests should be used and when they should be used for initial investigation	Blood tests discussed: routine blood tests, CA125 +/− CEA

Guidelines are grouped into categories on the bases of the number and order of tests advocated

Although the majority of guidelines used symptoms as the trigger for initiating tests, the two Australian short guides indicated that testing for ovarian cancer should be conducted if there was a suspicion on clinical examination [23, 24]. Conversely, guidelines from Ireland, England, Scotland, the UK, Sweden and Norway recommended that concerning findings on examination should prompt an urgent referral to a specialist rather than tests [18, 31–34, 37].

## Discussion

In the absence of effective screening programmes, most women are diagnosed with ovarian cancer following the onset of symptoms [10, 13]. In this review, we identified and compared international guidance documents on the initial assessment and investigation for possible ovarian cancer in symptomatic women. Our results highlight significant differences between international guidelines, not only in the clinical features they suggest should trigger a suspicion of ovarian cancer, but also in the initial examinations and investigations they advocate.

The stage distribution of ovarian cancer at diagnosis, and ovarian cancer survival, varies between countries [38]. A positive correlation has been demonstrated between national survival and the readiness of primary care practitioners to investigate or refer women with symptoms of possible ovarian cancer [39]. International variation in the way symptomatic women are assessed and investigated could also contribute to differences in the timeliness of ovarian cancer diagnosis and survival. Although guidelines are not always followed [40], they do influence practice [41, 42], and variation in international guidelines is likely to indicate differences in clinical practice internationally. International comparative research is ongoing to investigate differences in access to tests for ovarian cancer and survival [43]. Several studies have sought to evaluate the impact of national urgent cancer referral guidelines on timeliness of diagnosis and/or survival [42, 44, 45], but there is little research similarly evaluating the effect of guidelines which advocate symptom-triggered testing for ovarian cancer [46]. Studies are needed to evaluate the impact of such guidance to ensure that the recommended approaches are effective, for example, by comparing stage distribution and cancer survival pre- and post- implementation of guidance. Comparing the impact of cancer detection guidelines between countries is challenging, not least as it relies on the use of standardised endpoints (stage, survival) which are not always uniformly recorded. Initiatives such as the International Cancer Benchmarking Partnership [43], may improve consistency in the recording of such outcomes and so aid international comparisons.

Guideline developers have to consider the healthcare system for which they are developing guidance. The guidance from countries with National Health Services was, in general, specific on symptoms and signs and gave clear recommendations on which tests should be performed and in what order. In contrast, guidance from the USA, which has a Private Healthcare System, was much less prescriptive, providing different options for the clinician. This is likely to reflect the fact that National Health Services aim to provide uniform services and level of care across a country/region and must plan for this, while the care provided in a country with a Private Healthcare System may differ depending on the private provider. Similarly, guideline recommendations may be influenced by the speciality of the clinician performing the initial assessment within a healthcare system e.g. GP/family physician and/or gynaecologist. Gynaecologists may be more competent with, and willing to perform, gynaecological examinations and better equipped to interpret complex tests and algorithms. Direct access to gynaecologists is available in the USA and Germany and guidance from these countries included a range of specialist tests [47, 48]. In contrast, in countries like the UK, Ireland, Australia and Scandinavia, where GPs play a strong gatekeeping role and where a referral is generally required prior to gynaecology assessment, a limited number of tests were recommended.

Over the last 15 years a number of studies have explored associations between ovarian cancer and symptoms; however, differences exist between the symptoms they have identified and their predictive values. Most documents in this review included symptoms widely regarded as increasing the likelihood of an ovarian cancer being present, for example, abdominal distension and pelvic pain [4, 5, 49]. Some documents also included symptoms such as fatigue, nausea, back pain and the generic term ‘urinary symptoms’, which are more controversial, and were not found to increase the likelihood of ovarian cancer in a recent comprehensive systematic review [49]. Some variation may be due to the type of evidence that guideline developers chose to consider. For example, UK guideline developers appear to have taken account of all relevant international studies when deciding which symptoms should be included in the guidance [8]. In contrast, USA guidelines included a more restricted list of symptoms derived from the influential Ovarian Cancer Symptom Index which was developed in the USA [50]. As almost all published studies exploring associations between ovarian cancer and symptoms have been undertaken in the UK and the USA, guideline developers outside these countries must rely on international evidence to inform their recommendations [49]. Further large, high quality research studies, undertaken in countries around the world, would improve our understanding of the symptomology of ovarian cancer and help resolve disagreements over which symptoms should be included in guidelines.

Given the range of AGREEII scores guidelines obtained in the Rigour of Development domain, discrepancies in symptoms and other recommendations are likely stem in part from differences in the scope and quality of evidence reviews undertaken by guideline developers. It is likely that where a rigorous systematic approach is not followed, important research, for example on symptoms, may be missed. All guidance documents in this review are likely to influence patient care and should be developed rigorously and be explicit about the development process. Different strategies could help encourage this, which in turn could help to harmonise symptoms in international guidelines. For example, funders could have guidelines independently appraised following development, using the AGREEII checklist, and publish the results alongside the guidelines. In addition, many guidelines are published in peer reviewed journals. Guideline developers could be required to submit an AGREEII style checklist as part of the submission process. While not all guideline development groups have the significant resources required to develop all elements of clinical guidelines de novo, this may not be necessary. For example, the guidance from the New Zealand Guideline Group was based on 2005 NICE guidance and adapted to suit the New Zealand healthcare system. Collaboration by international guideline producers on aspects of guidelines such as symptoms, which are likely to differ little between healthcare systems or countries, could also help reduce duplication, ensure quality and increase consistency.

A pelvic or gynaecological examination was specifically recommended by half of the guidelines, with three specifying that a speculum and three a bimanual or digital examination, be performed. However, Myres et al.*’s* review, which included studies on examinations performed by gynaecologists pre-surgery and in the screening setting, found that less than half of adnexal masses are picked up on bimanual examination [51]. GPs might be less skilled at identifying pelvic masses, but a recent review identified no studies evaluating their competence at performing pelvic examinations for gynaecological cancer [52].

Most documents recommended the use of ultrasound and/or CA125 in the initial investigation for ovarian cancer. However, guidelines varied in the sequence of testing, and a variety of other serum biomarkers, imaging modalities and risk algorithms were included in some. This variation may result in part from differences in the funding and available resources within different healthcare systems. For example, consideration of costs and resource implications played a role in the decision by NICE to recommend the relatively cheap and widely accessible CA125 test rather than ultrasound as the first line investigation [8]. There is little high quality evidence for tests used in the initial investigation of possible ovarian cancer [8], often necessitating consensus opinion [34, 35], with one guideline making no recommendations on testing because of the lack of evidence [26].

Evidence from secondary care and screening studies indicates that CA125 and ultrasound differ in their diagnostic accuracy [8, 53, 54]. Therefore, the test(s) chosen, and, where they are used in combination, the order of testing, may have important implications for cancer detection. For example, a sequential testing approach, where both tests need to be abnormal to trigger specialist referral [33], will be more specific at the cost of lower sensitivity. Conversely, a dual-testing approach, where an abnormality in either test warrants referral [34, 35], will be more sensitive but sacrifices specificity and economy.

This is the first study to systematically identify and compare international guidance documents on the initial assessment and investigation for possible ovarian cancer in symptomatic women. Direct comparisons between the testing strategies employed in different countries must be interpreted with reference to the healthcare system for which the guidance was produced. Although we performed a comprehensive literature search, it is possible that we did not identify all relevant guidance documents e.g. healthcare guidelines not published online or not available outside the region or country of publication. We attempted to obtain all relevant documentation on the development process of guidelines included in this review, contacting guideline producers for additional information when necessary, to allow us to perform comprehensive AGREEII appraisals. However, it is possible that we did not gain access to all relevant documents e.g. unpublished search strategies or evidence reviews.

## Conclusion

Multiple international guidance documents provide advice on the initial assessment and investigation for possible ovarian cancer in symptomatic women. These documents differ markedly in the symptoms they include and the physical examinations and clinical investigations they recommend. Given this, it is probable that patient care and the likelihood of cancer detection will vary depending on the guidance document followed. Studies evaluating the role of examinations and the diagnostic performance of testing strategies for the initial assessment of possible ovarian cancer in symptomatic women are needed to aid the development of more evidence-based guidelines.

## Supplementary information

**Additional file 1: Figure S1.** Medline search strategy. **Table S1.** Scores in percent for each domain of guidance documents calculated using the AGREEII tool. **Table S2.** Summary of symptoms included in each guidance document. (DOCX 26 kb)

## Data Availability

This study was based entirely on previously published data which is available online from the sources described in the article. No datasets were developed or analysed in this study.

## Abbreviations

AGREEII: Appraisal for Guidelines and Research Evaluation 2
CT: Computed Tomography
MRI: Magnetic Resonance Imaging
NICE: National Institute of Health and Care Excellence
PPV: Positive Predictive Value
RMI: Risk of Malignancy Index
TRIP: Turning Research Into Practice
UK: United Kingdom
