# Supplementary material for: Variation in the initial assessment and investigation for ovarian cancer in symptomatic women: a systematic review of international guidelines
Source: BMC Cancer. 2019 Nov 1;19:1028. doi: 10.1186/s12885-019-6211-2 (PMC6823968; doi:10.1186/s12885-019-6211-2)
Supplement: Supplementary file 1 — Additional file 1: Figure S1. Medline search strategy. Table S1. Scores in percent for each domain of guidance documents calculated using the AGREEII tool. Table S2. Summary of symptoms included in each guidance document. (DOCX 26 kb) [file 12885_2019_6211_MOESM1_ESM.docx]

1 ovar*.mp. [mp=title, abstract, original title, name of substance word, subject heading word, keyword heading word, protocol supplementary concept word, rare disease supplementary concept word, unique identifier, synonyms]

2 OVARY/

3 cancer*.mp. [mp=title, abstract, original title, name of substance word, subject heading word, keyword heading word, protocol supplementary concept word, rare disease supplementary concept word, unique identifier, synonyms]

4 neoplas*.mp. [mp=title, abstract, original title, name of substance word, subject heading word, keyword heading word, protocol supplementary concept word, rare disease supplementary concept word, unique identifier, synonyms]

5 malignan*.mp. [mp=title, abstract, original title, name of substance word, subject heading word, keyword heading word, protocol supplementary concept word, rare disease supplementary concept word, unique identifier, synonyms]

6 Neoplasms/

7 1 or 2

8 3 or 4 or 5 or 6

9 7 and 8

10 limit 9 to ((consensus development conference or consensus development conference, nih or guideline or practice guideline) and last 10 years)

**Figure S1**. Medline search strategy.

**Table S1.** Scores in percent for each domain of guidance documents calculated using the AGREEII tool.

| **Guidance document** | **Scope and Purpose (%)** | **Stakeholder Involvement (%)** | **Rigour of Development (%)** | **Clarity of Presentation (%)** | **Applicability (%)** | **Editorial Independence (%)** |
| --- | --- | --- | --- | --- | --- | --- |
| **Continental Europe** | | | | | | |
| Epithelial ovarian carcinoma (Neth) | 83 | 69 | 66 | 72 | 40 | 58 |
| Guideline on diagnostics, therapy and follow-up of malignant ovarian tumours (Ger) | 83 | 69 | 81 | 92 | 13 | 96 |
| **United Kingdom and Republic of Ireland** | | | | | | |
| Epithelial ovarian / fallopian tube / primary peritoneal cancer guidelines: recommendations for practice (UK) | 50 | 36 | 48 | 56 | 10 | 42 |
| Ovarian cancer GP referral for symptomatic women (Ire) | 47 | 33 | 7 | 78 | 0 | 4 |
| Suspected cancer: recognition and referral (Eng) | 92 | 94 | 96 | 89 | 92 | 83 |
| Scottish referral guidelines for suspected cancer (Scot) | 86 | 92 | 55 | 83 | 33 | 4 |
| Management of epithelial ovarian cancer (Scot) | 86 | 83 | 76 | 92 | 60 | 79 |
| **Scandinavia** | | | | | | |
| Integrated ovarian cancer patient pathway (Den) | 78 | 64 | 29 | 89 | 90 | 0 |
| Ovarian cancer patient pathway (Nor) | 78 | 72 | 38 | 81 | 25 | 17 |
| Standardised ovarian cancer care pathway (Swed)^*^ | 100 | 81 | 55 | 92 | 35 | 46 |
| **Australasia** | | | | | | |
| Assessment of symptoms that may be ovarian cancer: a guide for general practitioners (Aus)^+^ | 75 | 81 | 50 | 72 | 38 | 25 |
| Appropriate referral of women with suspected ovarian cancer (Aus)^+^ | 75 | 81 | 50 | 72 | 38 | 25 |
| Optimal care pathway for women with ovarian cancer (Aus) | 64 | 64 | 10 | 78 | 13 | 13 |
| Suspected cancer in primary care: guidelines for investigation, referral and reducing ethnic disparity (NZ) | 83 | 75 | 56 | 72 | 35 | 75 |
| **North America** | | | | | | |
| Ovarian cancer: Including fallopian tube cancer and primary peritoneal cancer (USA) | 36 | 61 | 65 | 69 | 13 | 79 |
| The role of the obstetrician-gynaecologist in the early detection of epithelial ovarian cancer in women at average risk (USA) | 25 | 42 | 11 | 42 | 0 | 8 |
| Ovarian cancer diagnosis pathway map (Ont, Can) | 50 | 58 | 19 | 47 | 6 | 4 |
| Genital tract cancers in females: ovarian, fallopian tube, and primary peritoneal cancers (BC, Can) | 61 | 22 | 16 | 67 | 2 | 25 |

| AGREEII score | |
| --- | --- |
| >75% |  |
| 50-74% |  |
| 25-49% |  |
| <25% |  |

^*^AGREEII appraisal included an assessment of the full guideline evidence review.

^+^AGREEII assessment included appraisal of the rescinded 2004 clinical practice guideline on which these short guides are based.

**Table S2**

Summary of symptoms included in each guidance document

| **Symptom categories:**  **Guidelines** | **Abdomino-pelvic** | **Eating / appetite related** | **Urinary** | **Change in weight** | **Other** |
| --- | --- | --- | --- | --- | --- |
| **Continental Europe** | | | | | |
| Epithelial ovarian carcinoma (Neth) | - Vague gastrointestinal complaints  - Abdominal distension  - Bowel complaints | Nil | -Urinary complaints | Nil | Nil |
| Guideline on diagnostics, therapy and follow-up of malignant ovarian tumours (Ger) | *Occur repeatedly and persistently, particularly >50yrs:*  -Bloating  - Vague abdominal pain or discomfort | *Occur repeatedly and persistently, particularly >50yrs:*  - Feeling full | *Occur repeatedly and persistently, particularly >50yrs:*  - Increased frequency | Nil | Nil |
| **United Kingdom and Republic of Ireland** | | | | | |
| Epithelial ovarian / fallopian tube / primary peritoneal cancer guidelines: recommendations for practice (UK) | *Particularly when persistent for >1 year and occurring >12x/month:*  - Persistent abdominal distension  - Abdominal bloating  - pelvic or abdominal pain  - Change in bowel habit | *Particularly when persistent or for >1 year and occurring >12x/month:*  - Early satiety  - Loss of appetite | *Particularly when persistent or for >1 year and occurring >12x/month:*  - Increased urgency  - Increased frequency | - Unexplained weight loss | - Postmenopausal bleeding  - Fatigue |
| Ovarian cancer GP referral for symptomatic women (Ire) | *Persistent and frequent (i.e. >12x/month),* *especially if ≥50 years:*  - Abdominal distension  - Pelvic or abdominal pain  - New onset IBS  -Unexplained change in bowel habit | *Persistent and frequent (i.e. >12x/month),* *especially if ≥50 years:*  - Early satiety  - Loss of appetite | *Persistent and frequent (i.e. >12x/month),* *especially if ≥50 years:*  - Increasing urgency  - Increasing frequency | - Unexplained weight loss | *- Unexplained:*  - Fatigue  - DVT |
| Suspected cancer: recognition and referral (Eng)^*^ | *Persistent or frequent (particularly if > 12x /month), especially if >50 years:*  - Persistent abdominal distension (bloating)  - Pelvic / abdominal pain  >50 years with symptoms of IBS in last 12 months  - Changes in bowel habit | *Persistent or frequent (particularly if > 12x /month), especially if >50 years:*  - Feeling full (early satiety)  - Loss of appetite | *Persistent or frequent (particularly if > 12x /month), especially if >50 years:*  - Increased  urgency  - Increased frequency | - Unexplained weight loss | - Fatigue |
| Scottish referral guidelines for suspected cancer (Scot)^*^ | *Unexplained and recurrent (most days), especially >50 years:*  - Abdominal distension or persistent bloating  - Pelvic or abdominal pain  - Change in bowel habit  >50 years, new symptoms suggestive of IBS in last 12 months | *Unexplained and recurrent (most days), especially >50 years:*  - Feeling full quickly  - Difficulty eating  - Loss of appetite | *Unexplained and recurrent (most days), especially >50 years:*  - Increased urgency  - Increased frequency | Nil | Nil |
| Management of epithelial ovarian cancer (Scot) | *Less than 12 months duration, occurring >12x/month:*  - Abdominal distension or bloating  - Abdominal pain | *Less than 12 months duration, occurring >12x/month:*  - Feeling full quickly  - Difficulty eating | *Less than 12 months duration, occurring >12x/month:*  - Urinary symptoms | Nil | Nil |
| **Scandinavia** | | | | | |
| Integrated ovarian cancer patient pathway (Den) | *Persistent for >1 month:*  - Abdominal bloating / distension  - Constipation  -Ileus / Sub-ileus | *Persistent for >1 month:*  - Loss of appetite  - Nausea | *Persistent for >1 month:*  - Urinary frequency | Nil | *Persistent for >1 month:*  - Dyspnoea  -Fatigue |
| Ovarian cancer patient pathway (Nor) | - Abdominal bloating  - Feeling of pressure  -Increased abdominal size  - Change in bowel habit e.g. diarrhoea or constipation  - Acute pain | - Loss of appetite | - Urinary frequency | - Unexpected weight loss  - Unexpected weight gain | - General symptoms  - Dyspnoea  - Vaginal bleeding  - Symptoms of DVT in lower extremity  - Paraneoplastic phenomenon |
| Standardised ovarian cancer care pathway (Swed)^+^ | *New:*  - Increased pelvic or abdominal size  - Pelvic or abdominal pain  - New IBS in women >50yrs  - Abdominal pressure symptoms  - Change in bowel habit (unexplained) | *New, unexplained:*  - Loss of appetite  - Early satiety | *New:*  - Frequency  - Urgency | Nil | *New, unexplained:*  - Symptoms of DVT in lower extremity |
| **Australasia** | | | | | |
| Assessment of symptoms that may be ovarian cancer: a guide for general practitioners (Aus) | *Persistent for >1 month:*  - Abdominal bloating  - Increased abdominal girth  - Change in bowel habit  - Abdominal and / or pelvic pain  - Feeling of pressure in the abdomen | *Persistent for >1 month:*  - Lack of appetite  - Feeling full after only a small amount of food  - Indigestion | Persistent for >1 month:  - Frequency  - Incontinence | *Persistent for >1 month:*  - Weight gain  - Weight loss | *Persistent for >1 month:*  - Fatigue |
| Appropriate referral of women with suspected ovarian cancer (Aus) | *Persistent for >1 month:*  - Abdominal bloating  - Constipation  - Abdominal / pelvic pain | *Persistent for >1 month:*  - Feeling full  - Appetite loss  - Heartburn | *Persistent for >1 month:*  - Frequency | *Persistent for >1 month:*  - Unexpected weight loss | *Persistent for >1 month:*  - Back pain  - Fatigue |
| Optimal care pathway for women with ovarian cancer (Aus) | *Persist for >1month, particularly in older women and those with family history:*  - Abdominal bloating  - Increased abdominal girth  - Abdominal and /or pelvic pain  - Change in bowel habit  - Feeling pressure on the abdomen | *Persist for >1month, particularly in older women and those with family history:*  -Indigestion  - Lack of appetite  - Feeling full after only a small amount of food | *Persist for >1month, particularly in older women and those with family history:*  - Frequency  - Incontinence | *Persist for >1month, particularly in older women and those with family history:*  - Weight gain  - Weight loss | *Persist for >1month, particularly in older women and those with family history:*  - Fatigue |
| Suspected cancer in primary care: guidelines for investigation, referral and reducing ethnic disparity (NZ)^*^ | *Unexplained:*  - Bloating  - Constipation  - Abdominal pain | Nil | - Unexplained urinary symptoms | Nil | - Unexplained back pain |
| **North America** | | | | | |
| Ovarian cancer: Including fallopian tube cancer and primary peritoneal cancer (USA) | *Without a source of malignancy:*  - Bloating  - Pelvic / abdominal pain | *Without a source of malignancy:*  - Difficulty eating  - Feeling full quickly | *Without a source of malignancy:*  - Urgency  - Frequency | Nil | Nil |
| The role of the obstetrician-gynaecologist in the early detection of epithelial ovarian cancer in women at average risk (USA)^^^ | *More than 12 days per month of new onset (less than 12 months’ duration):*  - Increase in abdominal size / bloating  - Pelvic or abdominal pain | *More than 12 days per month of new onset (less than 12 months’ duration):*  - Difficulty eating  - Feeling full quickly | Nil | Nil | Nil |
| Ovarian cancer diagnosis pathway map (Ont, Can) | *-Persistent and / or unexplained:*  -Pelvic or abdominal pain  -Gastrointestinal symptoms e.g. bloating  -Increased abdominal size | -Difficulty eating (early satiety, nausea) | *-Persistent and / or unexplained:*  -Urgency  -Frequency | Nil | -Abnormal vaginal bleeding |
| Genital tract cancers in females: ovarian, fallopian tube, and primary peritoneal cancers (BC, Can) | *Lasting 2-3 weeks:*  - Persistent abdominal distension / bloating  - Abdominal discomfort or pain  - Change in bowel habits  - Pelvic pressure | *Lasting 2-3 weeks:*  - Early satiety  - Heartburn | *Lasting 2-3 weeks:*  - Frequency  - Nocturia | Nil | *Lasting 2-3 weeks:*  - Postmenopausal / abnormal bleeding  - Unexplained thromboembolism |

Where there are discrepancies between the symptoms mentioned in the body of the text and those included in ‘recommendations’ section of guidelines, those included in the recommendation section are shown.

^*^Guideline covers multiple cancers. Other symptoms of potential relevance e.g. abnormal vaginal bleeding, may be discussed in sections relating to other cancers e.g. endometrial cancer.

^+^Both a full clinical practice guideline covering initial assessment, definitive diagnosis and treatment, and a short version focussing on initial assessment and investigation in primary care are available. Guidance on initial assessment differed slightly between the two documents. The presented data was extracted from the short guide.

^^^Recommendation section states “maintain an appropriate level of suspicion when potentially relevant signs and symptoms of ovarian cancer are present”. Symptoms listed are highlighted later in the document in a separate symptoms and signs section.
